# Walnut Green Husk Extract Enhances Antioxidant, Anti-Inflammatory, and Immune Functions by Regulating Gut Microbiota and Metabolites in Fattening Pigs

**DOI:** 10.3390/ani15162395

**Published:** 2025-08-15

**Authors:** Jing Wang, Mingyang Jia, Qi Zhang, Xiangzhou Yan, Yaping Guo, Lei Wang, Baosong Xing

**Affiliations:** 1 Henan Key Laboratory of Farm Animal Breeding and Nutritional Regulation, Henan Pig Breeding Engineering Research Centre, Institute of Animal Husbandry, Henan Academy of Agricultural Sciences, Zhengzhou 450002, China; wangjing@hnagri.org.cn (J.W.); mingyangjia@hnagri.org.cn (M.J.); zhangqi0230@163.com (Q.Z.); xiangzhouyan@hnagri.org.cn (X.Y.); ypguo525@163.com (Y.G.); 2 College of Animal Science and Veterinary Medicine, Henan Institute of Science and Technology, Xinxiang 453003, China

**Keywords:** fattening pig, walnut green husk extract, 16s rDNA sequencing, untargeted metabolomics, functional feed additive

## Abstract

This study investigates the effect of walnut green husk extract (WE) on gut microbiota, metabolites, and immune-antioxidant changes in fattening pigs through gut microbiota-metabolite interactions. A total of 60 healthy fattening pigs (Duroc × Landrace × Yorkshire) with an initial body weight of 65.2 ± 3.1 kg were randomly assigned to two groups (n = 30 per group): the control group (NC), which was fed a basal diet, and the WE group, which was fed the basal diet supplemented with 0.1% walnut green husk extract (WE). Dietary supplementation with 0.1% WE significantly increased the relative abundances of beneficial bacteria (e.g., *Firmicutes*, *Lactobacillus*) and reduced pathogenic bacteria (e.g., *Proteobacteria*, *Shigella*). Untargeted metabolomics identified 170 differentially accumulated metabolites, among which propionic acid—a key short-chain fatty acid with immunomodulatory effects—was significantly upregulated by 1.09-fold (*p* = 0.03) and showed a positive correlation with beneficial microbial abundances. These metabolites were enriched in glycerophospholipid and α-linolenic acid metabolism pathways, where eicosadienoic acid inhibited the nuclear factor kappa-B (NF-κB) pathway for anti-inflammatory effects, and methyl cinnamate synergistically regulated mitogen-activated protein kinase (MAPK) signaling with *Lactobacillus*. Serum analyses showed that WE significantly enhanced IgA, IgM, and IgG levels by 3.97-fold, 4.67-fold, and 4.43-fold (*p* < 0.01), reduced malondialdehyde (MDA) concentration by 82.8% (*p* < 0.01), and trended to improve antioxidant capacity via glutamine. Mechanistically, WE promoted short-chain fatty acid production by beneficial bacteria, forming a “microbiota–metabolite–immunity” cascade to enhance lipid metabolism and alleviate intestinal inflammation. These findings highlight that WE provides multi-omics evidence for its application as a functional feed additive.

## Simple Summary

Currently, the dual pressures of rising feed raw material prices and restricted antibiotic use in feed have created an urgent need for antibiotic alternatives that can also improve breeding efficiency. This study analyzed the water extract of walnut green husk, examining its antioxidant, anti-inflammatory, and immune-enhancing effects on fattening pigs. Results showed it regulates gut microbiota, promoting beneficial bacteria and inhibiting harmful ones, while altering 170 metabolites—some with anti-inflammatory effects that synergize with beneficial bacteria to maintain intestinal health. Pigs receiving the extract exhibited 3.97–4.67-fold higher serum levels of IgA, IgM, and IgG, an 82.8% reduction in the cell damage marker MDA, enhanced lipid metabolism, and reduced intestinal inflammation. Mechanistically, the extract promotes short-chain fatty acid production by beneficial bacteria, forming a “gut microbiota–metabolite–immunity” cascade that improves lipid handling and reduces intestinal inflammation. As an antibiotic alternative with breeding efficiency benefits, it enables waste recycling and supports healthy, economical, and sustainable animal husbandry.

## 1. Introduction

In recent years, global food prices have fluctuated considerably, with especially high increases in the prices of corn, soybean meal, and other major raw materials for feed, which has directly led to increases in feed costs. With the implementation of the policy in prohibition of antibiotics use for feed, livestock and poultry diseases occur frequently. Therefore, there is an urgent need to find eco-friendly natural additives that can improve feed utilization and replace antibiotics.

Plant extracts (PEs), as a class of natural bioactive substances with broad application potential, have garnered significant attention in the field of animal nutrition and health in recent years. Their unique chemical composition endows them with a variety of biological functions, including antimicrobial, anti-inflammatory, antioxidant, and immunomodulatory properties, making them ideal candidates to replace antibiotic growth promoters (AGPs). Studies indicate that plant extracts (PE) boost animal health and productivity by inhibiting pathogens, enhancing intestinal barriers, and balancing gut microbiota. For example, Meligy et al. [1] found that liposomal oregano, cinnamon, and clove oils increased beneficial bacteria and their metabolites (e.g., short-chain fatty acids) while reducing pathogens in broilers. Cui et al. [2] showed tartary buckwheat flavonoids elevated Bacteroidetes in piglets, and combined with Lactobacillus plantarum, increased Mitsuokella (linked to combating digestive disorders [3]). Lv et al. [4] noted genistein in broiler diets stimulated immune cell activity and raised IgM, IgG, antibody potency, and antioxidant capacity. Additionally, the addition of mustard seed extract to drinking water significantly increased the body weight of broilers while reducing the feed-to-weight ratio [5]. In summary, PEs, with their multifunctional biological properties, demonstrate significant application potential in improving animal health, enhancing production performance, and promoting the development of sustainable animal husbandry.

Walnut (*Juglans regia* L.) is one of the four major nuts widely cultivated worldwide. The green husk, the outer layer of walnuts, is rich in various bioactive substances such as polysaccharides, polyphenols, flavonoids, terpenoids, and naphthoquinones; among these, polysaccharides are the main component, accounting for up to 72.4% (with the highest reported content exceeding 70%) [6,7]. Previous studies have confirmed that plant polysaccharides exhibit diverse biological activities, including immunomodulation, antioxidant effects, and regulation of gut microbiota [8]. Additionally, walnut green husk extract (WE) has been found to inhibit *Escherichia coli* [9] and *Staphylococcus aureus* [10], which provides a theoretical basis for its application in the feed field. However, research on WE as a feed additive is currently limited. A study by Wu et al. showed that adding WE to the diet of broilers did not improve growth performance, feed conversion efficiency, slaughter performance, or immune organ indices [11]. Nevertheless, it could reduce abdominal fat deposition, improve meat tenderness and antioxidant status, maintain intestinal villus integrity, and increase gut microbiota diversity. Our previous study also found that adding 0.1% WE to the diet of finishing pigs significantly increased meat quality traits such as intramuscular fat content (+98.59%, *p* < 0.05) and backfat thickness (+33.12%, *p* < 0.05). Meanwhile, growth traits including average daily gain (+4.88%, *p* > 0.05), carcass weight (+4.66%, *p* > 0.05), and dressing percentage (+4.82%, *p* > 0.05) showed an upward trend [12]. Given the high polysaccharide content in WE, we hypothesize that polysaccharides may be the key active components responsible for its effects. They might regulate the antioxidant capacity, inflammatory response, and immune function of finishing pigs, potentially through modulating the interactions between gut microbiota and metabolites. To further clarify this underlying mechanism, this study employed 16S rDNA sequencing, metabolome sequencing, and ELISA to analyze the effects of WE supplementation on fecal microbiota, fecal metabolites, immune function, antioxidant capacity, and serum biochemical indicators in castrated male finishing pigs. The aim is to provide basic data for alleviating the shortage of feed resources and developing new functional feeds.

## 2. Materials and Methods

### 2.1. Animal Ethics

Slaughter was conducted in line with standard commercial protocols: electrical stunning was performed at 220 V, 1.5 A for 5 s, and exsanguination was carried out through an incision in the carotid artery, in compliance with GB/T 42304-2023 [13]. All the procedures received approval from the Institutional Animal Care and Use Committee of Henan Academy of Agriculture Sciences (Zhengzhou, China, ID: 2023-8[1]).

### 2.2. Experimental Materials

WE was purchased from Xi’an Osai Biotechnology Co., Ltd. (Xi’an, China). The extract was prepared via a standardized water extraction process optimized for yield and bioactive components: walnut green husk powder was mixed with deionized water at a 10:1 (*w*/*v*) ratio, extracted at 80 °C for 2 h under magnetic stirring, filtered through a 0.45-μm membrane, and concentrated by rotary evaporation at 50 °C under reduced pressure. The concentrate was spray-dried (inlet temperature 180 °C, outlet temperature 90 °C) to obtain a fine powder. The protein content (1.06 g/100 g, determined by the Kjeldahl method), polysaccharide content (72.4 g/100 g, measured by the phenol-sulfuric acid method), total flavonoids (0.07% expressed as quercetin equivalents), and total phenols (4.79 mg/g as gallic acid equivalents) were quantified using standardized assays.

### 2.3. Laboratory Animals, Diet, and Husbandry Management

In this study, Duroc × (Landrace × Yorkshire) ternary crossbred castrated finishing male pigs with an average initial body weight of 65.2 ± 3.1 kg were used as experimental animals. The feeding trials were conducted at the Xinmi Breeding Base of the Institute of Animal Husbandry, Henan Academy of Agricultural Sciences. Using a completely randomized design (CRD), 60 pigs were randomly allocated into 2 groups with 3 replicates per group (10 pigs per replicate). Each replicate was housed separately in pens divided by fences. The experimental groups were as follows: (1) basal diet (negative control group); (2) basal diet supplemented with 0.1% WE (WE group). The composition and nutrient levels of the basal diet are presented in Table 1. The 0.1% WE was incorporated into the basal diet through thorough mixing: the WE powder was weighed according to the proportion of the basal diet, added to the basal diet in small batches, and mixed uniformly using a feed mixer for 15 min to ensure homogeneous distribution. The mixed feed was stored in clean, sealed containers at room temperature (20–25 °C) and used within 3 days to avoid moisture absorption or degradation of bioactive components. Given the average daily feed intake of fattening pigs (approximately 2.5 kg per pig per day), the actual daily intake of WE per pig in the WE group was 2.5 g, which corresponds to the 0.1% supplementation level.

The experimental pig house was a traditional semi-open structure, which was thoroughly cleaned and sterilized prior to the experiment. All pigs were provided with ad libitum access to feed and water, and the use of antibiotics or other medications was prohibited. Feed and water were replenished daily at 09:00 and 16:00. A 10-day pre-feeding period was implemented before the start of the experiment, during which all pigs exhibited normal feed intake without signs of physiological stress. The formal experimental period, during which pigs in the WE group were continuously fed the basal diet supplemented with 0.1% walnut green husk extract (WE) and those in the NC group were fed the basal diet without WE, lasted 40 days (from Day 10 to Day 50, following the 10-day pre-feeding period). Regular disinfection was performed. At the end of the experiment, one pig was randomly selected from each of the 3 replicates in both the NC and WE groups (3 pigs per group in total) for slaughtering. To ensure representativeness, the selected pigs were matched to the average body weight of their respective pens (*p* > 0.05). Fresh fecal samples were collected, immediately frozen in liquid nitrogen for 12 h, and then transferred to −80 °C for storage. Blood samples were collected from the jugular vein prior to slaughter. Euthanasia was performed in accordance with GB/T 42304-2023 to obtain carcass and meat quality traits (e.g., intramuscular fat content, backfat thickness), which were reported in our previous study [12]. Fecal and blood samples were concurrently collected during this process to investigate gut microbiota, metabolites, and systemic indices, ensuring consistency with the overall experimental design. The experimental period, housing conditions, and diet formulation were consistent with our previous study [12], where growth performance traits (e.g., average daily gain, carcass weight, dressing percentage) were reported. The current study focused on gut microbiota, metabolites, and serum indices, with sample collection synchronized with the growth performance evaluation in previous study.

### 2.4. Fecal Microbiota Analysis

To evaluate the regulatory effect of WE supplementation—specifically, its impact on altering the composition, relative abundance, and diversity of the intestinal microbial community (including changes in beneficial and pathogenic bacteria) in pigs, this study conducted 16S rDNA gene sequencing analysis on fecal samples collected from experimental and control groups. Genomic DNA extraction was performed using a modified CTAB method, with the lysis buffer formulated as 2% (*w*/*v*) cetyltrimethylammonium bromide, 100 mM Tris-HCl buffer (pH 8.0), 1.4 M sodium chloride, 20 mM ethylenediaminetetraacetic acid, and 1% (*w*/*v*) polyvinylpyrrolidone. DNA concentration and purity were detected using a Qubit 3.0 fluorometer, requiring the A260/A280 ratio to be within the range of 1.8–2.0. Integrity verification was achieved through 1% agarose gel electrophoresis (90 V constant voltage, 45 min), with the qualification criteria being clear single bands and no diffuse smearing.

Amplification of the V3-V4 variable region was carried out using primer combinations with sample-specific barcodes (forward primer 341F: 5′-CCTACGGGNGGCWGCAG-3′; reverse primer 805R: 5′-GACTACHVGGGTATCTAATCC-3′). The PCR reaction system (20 μL) contained 4 μL of 5× FastPfu buffer, 2 μL of 2.5 mM dNTP mixture, 0.8 μL of each upstream and downstream primer (final concentration 0.2 μM), 0.4 μL of FastPfu DNA polymerase, and 10 ng of template DNA. The amplification program was set as follows: pre-denaturation at 95 °C for 2 min; denaturation at 95 °C for 30 s, annealing at 55 °C for 30 s, extension at 72 °C for 60 s, for 27 cycles; final extension at 72 °C for 5 min. Each sample was set with 3 technical replicates, and product quantification was performed using the QuantiFluor™-ST blue fluorescence quantification system.

During library construction, 50 ng of purified PCR product was directly used for SMRTbell template preparation. Since the target fragment length (approximately 400–500 bp) already met the sequencing requirements, the DNA fragmentation step was omitted. Two rounds of purification were performed using AMPure XP beads to strictly screen target fragments in the 400–500 bp range, effectively removing primer dimers and non-specific amplification products. The sequencing on the PacBio Sequel II platform used a single SMRT Cell. After sequencing, data processing was performed using SMRTLINK v11 software to generate circular consensus sequences (CCS), with quality filtering parameters set as: minimum number of passes ≥ 3, sequence quality value QV ≥ 20. The obtained high-quality sequences had an average read length of approximately 450 bp, with an average of 48,318 effective reads per sample.

The bioinformatics analysis process was as follows: first, CCS were generated based on SMRTLINK (v11); then, OTU clustering was performed using the VSEARCH (v2.14.2) algorithm at 98.65% sequence similarity, while chimera detection and filtering were completed through the UCHIME (v8.1) algorithm. Microbial community composition analysis and α/β diversity evaluation were conducted based on OTU classification results. Functional prediction analysis used PICRUSt2 (version 2.1.4) software for KEGG pathway annotation based on the Greengenes 2020 database. Before analysis, normalization correction was performed on 16S rDNA gene copy numbers to eliminate deviations caused by differences in gene copy numbers among different genera.

### 2.5. Untargeted Metabolomics Analysis

The collected fecal samples were subjected to metabolomic analysis. Metabolites were extracted using a pre-chilled methanol–water solution (volume ratio 4:1, 500 μL of extraction solution corresponding to each 100 mg of tissue sample), with internal standard substances [such as L-2-chlorophenylalanine (10 μM) and deuterated glucose (d7-glucose, 50 μM)] added to the system. After the samples underwent vortex oscillation for 2 min, ultrasonic disruption for 15 min under ice-bath conditions, and centrifugation at 14,000× *g* for 20 min in a low-temperature environment at 4 °C, the supernatant was collected and subjected to vacuum freeze-drying treatment. Before LC-MS analysis, the freeze-dried metabolites were reconstituted with a 10% methanol solution (100 μL). Quality control (QC) samples were prepared by mixing equal-volume samples of all biological extracts, and a solvent blank group was set up simultaneously to monitor background interference. QC samples were processed in sync with experimental samples, and injection was performed once for every 10 samples to evaluate the reproducibility of the analysis process.

Ultra-high-performance liquid chromatography (UHPLC) separation was completed with the aid of a Vanquish UHPLC system, equipped with a Hypesil Gold chromatographic column (1.9 µm particle size, 2.1 × 100 mm specification), and coupled with a Q Exactive™ HF-X mass spectrometer. The data-independent acquisition (DIA) mode was adopted to simultaneously collect mass spectrometry data in both positive and negative ion modes. Progenesis QI software (v2.3) was used for the identification, correction, and normalization processing of metabolite features. Orthogonal partial least squares discriminant analysis (OPLS-DA) was carried out via metaX v1.0 software to optimize intergroup discrimination and screen for differentially accumulated metabolites (DAM) between the NC and WE groups. The feature detection parameters were set as follows: retention time tolerance of 0.2 min and mass deviation tolerance of 5 ppm. DAMs were screened using VIP ≥ 1, *p*-value < 0.05, and FDR < 0.05 as criteria, combined with |log2FC| ≥ 1. Data normalization adopted the total ion current (TIC) normalization method, and missing values were filled by means of adding 10% of the standard deviation to the minimum value of each feature. The OPLS-DA model employed a 7-fold cross-validation strategy, and 200 permutation tests were conducted to evaluate the model reliability. The results showed a permutation *p* value of 0.01, an R^2^Y intercept of 0.12, and a Q^2^ intercept of −0.35. Finally, the KEGG database and Human Metabolome Database (HMDB) were utilized for metabolic pathway enrichment analysis.

### 2.6. Measurement of Serum Immunity Indices, Antioxidant Indices, and Lipid Indices

Immunoglobulin A (IgA), IgG, IgM, superoxide dismutase (SOD), glutathione peroxidase (GSH-Px), catalase (CAT), malondialdehyde (MDA), and total antioxidant capacity (T-AOC), total cholesterol (TC), triglycerides (TG), high-density lipoprotein cholesterol (HDL-C), and low-density lipoprotein cholesterol (LDL-C) were measured using a Hitachi Automatic Analyzer 7600 (Tokyo, Japan) with ELISA kits provided by Jiancheng Biological Institute (Nanjing, China). Each serum sample was analyzed in triplicate as technical replicates; for each biological replicate (i.e., individual animal), three parallel measurements were performed to assess assay reproducibility, and the mean value was used for subsequent statistical analysis.

### 2.7. Statistical Analysis

The statistical significance of the differences was evaluated using SPSS 22.0 software, and the results were reported as the means ± SEMs. Comparisons between the two groups were performed using *t*-tests. Differences were considered significant when * *p* < 0.05 or ** *p* < 0.01.

## 3. Results

### 3.1. Diversity of Fecal Microbiota

**Figure 1:**
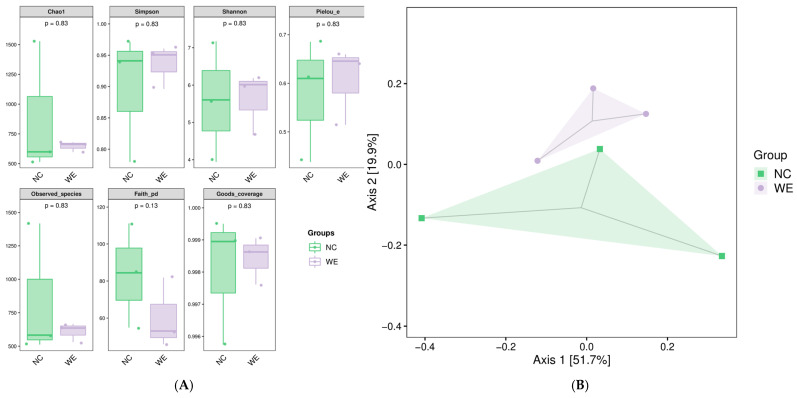
Effects of dietary walnut green husk extract on fecal microbial diversity in fattening pigs. (**A**) Boxplots showing the results of alpha diversity analysis, including the Chao1 index, Shannon index, Simpson index, Pielou_e index, Observed_species index, Faith_pd index, and Good_coverage index, in the NC and WE groups. No significant differences were observed between the two groups (*p* > 0.05). (**B**) Scatter plot of principal coordinate analysis (PCoA) based on weighted UniFrac distance. Axis 1 (explained variance: 51.7%) and Axis 2 (explained variance: 19.9%).

To assess the diversity of the microbial community, each sample was analyzed using alpha diversity analysis. The results, as shown in Figure 1A, indicated no significant changes in the Chao1 index, Simpson index, Shannon index, Pielou_e index, Observed_species index, Faith_pd index, or Goods_coverage index between the NC and WE groups (*p* > 0.05). In the Beta diversity analysis, PCoA based on weighted Bray–Curtis distance showed a moderate degree of difference in the microbial community structures of the two groups (Figure 1B). The Adonis test (Table 2) did not show statistical significance (*p*-value = 1.00). 

### 3.2. Composition of the Fecal Microbiota

**Figure 2:**
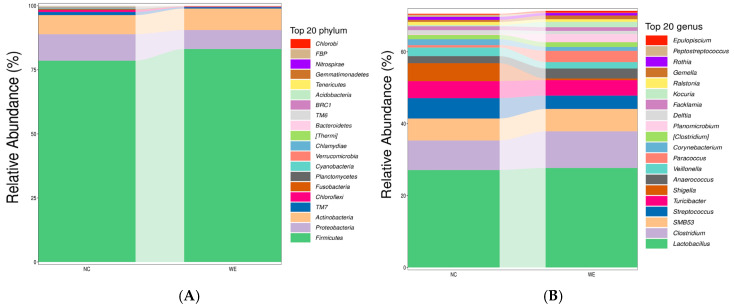
Effects of dietary walnut green husk extract on the composition of fecal microbiota in fattening pigs. (**A**) Bar plot showing the relative abundance of the top 20 phyla in the NC and WE groups at the phylum level. (**B**) Bar plot showing the relative abundance of the top 20 genera in the NC and WE groups at the genus level.

In this study, 28 phyla and 443 genera were identified in the NC group, while 21 phyla and 343 genera were identified in the WE group (detailed data on the relative abundances of phyla and genera in both groups are provided in Supplementary Tables S1 and S2). At the phylum level, the relative abundance of *Firmicutes* and *Actinobacteria* increased, whereas the relative abundance of *Proteobacteria* decreased compared to the NC group (Figure 2A). At the genus level, the relative abundance of *Lactobacillus* and *Clostridium* increased, while the relative abundance of *Streptococcus*, *Turicibacter*, and *Shigella* decreased compared to the NC group (Figure 2B).

### 3.3. LEfSe Analysis of Fecal Microbiota

**Figure 3:**
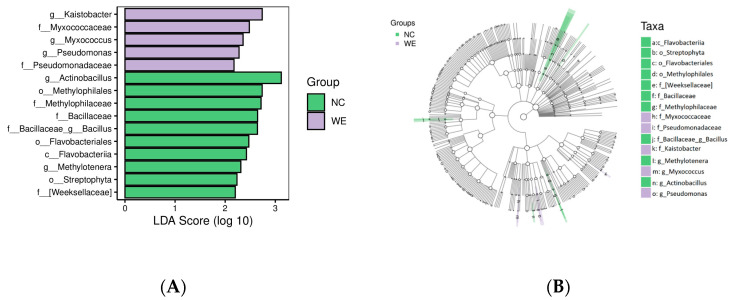
Analysis of differential microbiota between the NC and WE groups. (**A**) Bar graph of LDA effect values. (**B**) Branching diagram of LEfSe analysis.

Differences were analyzed at all taxonomic levels using LEfSe (Linear Discriminant Analysis Effect Size) analysis. Figure 3 shows the results of LEfSe analysis of the fecal microbiota in the NC and WE groups, with the LDA threshold set at 2.0. A total of 15 bacterial taxa showed significant differences between the two groups. The results indicated that *g_Kaistobacter*, *f_Myxococcaceae*, *g_Myxococcus*, *g_Pseudomonas*, and *f_Pseudomonadaceae* were the representative taxa in the WE group.

### 3.4. Metabolic Profiling

**Figure 4:**
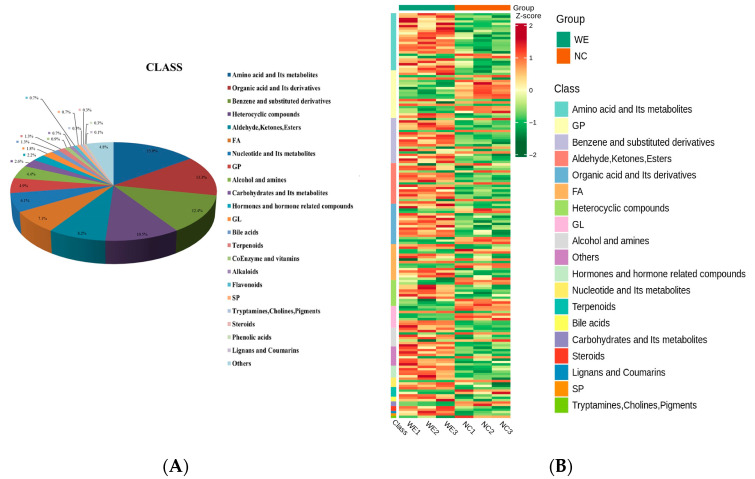
Composition (**A**) and clustering analysis heatmap (**B**) of metabolites in feces from NC and WE groups. Colors correspond to the distinct values achieved following relative content normalization (red denotes high levels and green denotes low levels). Note: Due to rounding, the total percentage may slightly deviate from 100%.

After metabolome sequencing, a total of 2509 metabolites were identified, including 1585 metabolites in positive-ion mode and 924 metabolites in negative-ion mode (Table S3). The metabolites from both ion modes were combined and categorized. Based on their secondary classification, the metabolites were primarily grouped into the following classes: 286 amino acids and their metabolites (15.0%), 254 organic acids and their derivatives (13.3%), 238 benzene and substituted derivatives (12.4%), 200 heterocyclic compounds (10.5%), 156 aldehydes, ketones, and esters (8.2%), 136 FAs (7.1%), 117 nucleotides and their metabolites (6.1%), and 94 GPs (4.9%) (Figure 4A). Differences in metabolomics profiles were visualized using a cluster analysis heatmap (Figure 4B).

### 3.5. Differentially Accumulated Metabolite Analysis

**Figure 5:**
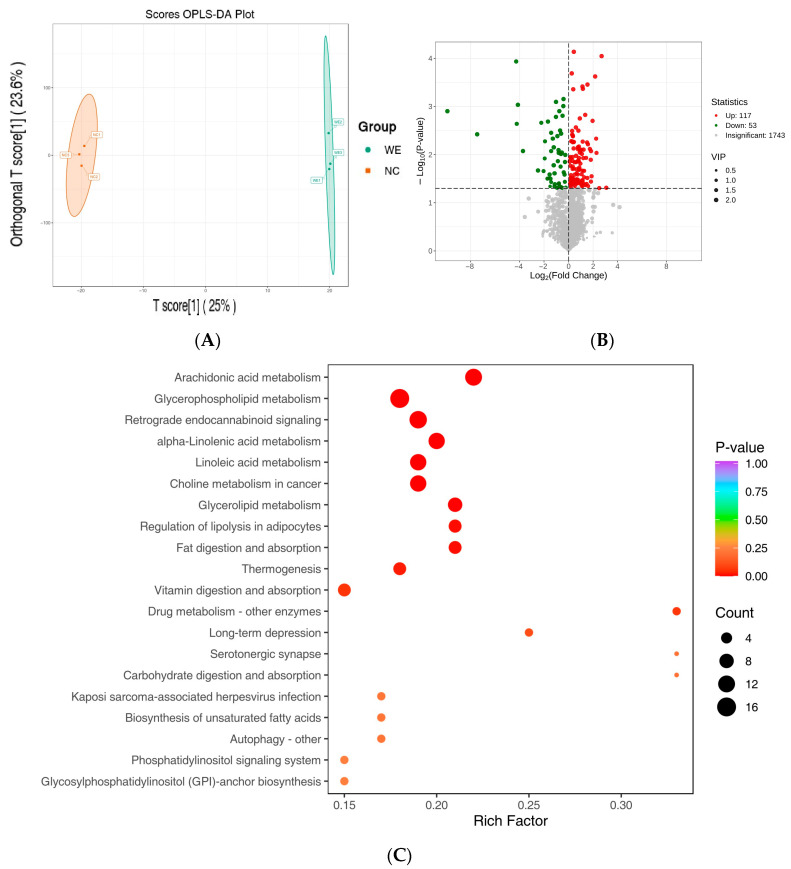
Investigation of differentially accumulated metabolites between the NC and WE groups. (**A**) Fecal metabolite profiling was performed using OPLS-DA models between NC and WE groups. (**B**) Volcano graphs showing the differentially accumulated metabolites between NC and WE groups. The *x*-axis signifies the Rich Factor associated with each pathway, while the *y*-axis shows the names of the pathways arranged in order of their *p*-value. The red circles show the upregulated metabolites; the green circles show the downregulated metabolites. (**C**) The KEGG enrichment plots show the metabolic pathways enriched with specific metabolites that are expressed differently between the NC and WE groups. The color of the data points reflects the size of the *p*-value, where red shades suggest a higher level of enrichment. The magnitude of the data points corresponds to the quantity of metabolites that are differentially expressed and enriched in that particular pathway.

In the OPLS-DA model (Figure 5A), the WE group was clearly separated from the NC group, indicating significant differences in fecal metabolite profiles between the two groups. Differentially accumulated metabolites (DAMs) were screened using VIP > 1 *p*-value < 0.05 and FDR < 0.05 as criteria. The characteristics of the screened differential metabolites were visualized in a volcano plot (Figure 5B), and a total of 170 DAMs (117 upregulated and 53 downregulated) were identified (Tables S4 and S5). Key differential metabolites are shown in Table 3. Among these, amino acids and their metabolites, GPs, and benzene and substituted derivatives accounted for a significant proportion. Specific metabolites such as PC (18:3/18:3), propionic acid, eicosadienoic acid, methyl cinnamate, and others were significantly upregulated.

### 3.6. Metabolic Pathway Analysis

Pathway enrichment analysis of the differential metabolites was performed using the KEGG database. The top 20 pathways, ranked by *p*-value from smallest to largest, are shown in Figure 5C. These differential metabolites were significantly associated with arachidonic acid metabolism, glycerophospholipid metabolism, alpha-linolenic acid metabolism, linoleic acid metabolism, choline metabolism, glycerolipid metabolism, regulation of lipolysis, fat digestion and absorption, and other metabolic pathways.

### 3.7. Effects of WE on Immunological Indicators in Fattening Pigs

**Figure 6:**
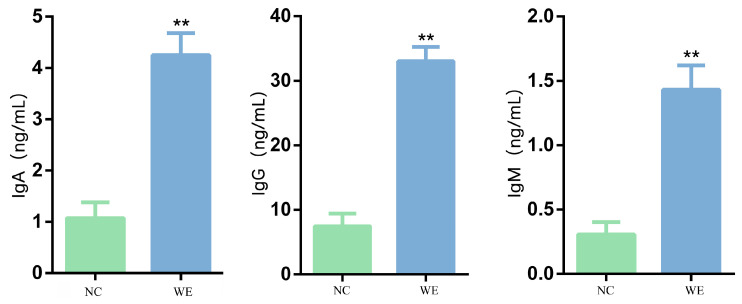
Effect of dietary supplementation with walnut green husk extract on serum immunity index in fattening pigs. ** mean extremely significant difference (*p* < 0.01).

The addition of WE to the diet highly significantly increased the levels of IgA (+3.97-fold, *p* < 0.01), IgG (+4.67-fold, *p* < 0.01), and IgM (+4.43-fold, *p* < 0.01) in fattening pigs compared to the NC group (Figure 6).

### 3.8. Effects of WE on Antioxidant Activity in Fattening Pigs

The addition of WE to the diet highly significantly reduced MDA concentration (−82.84%, *p* < 0.01) compared to the NC group (Table 4). While the activities of serum SOD, GSH-Px, CAT, and T-AOC (*p*-value = 0.09) showed increasing trends in the WE group, these changes did not reach statistical significance (*p* > 0.05) (Table 4).

### 3.9. Effects of WE on Lipid Indices in Fattening Pigs

The addition of WE to the diet tended to increase serum TG (+36.00%) and TC (+11.64%) levels and decrease serum LDL-C levels (−15.49%) in fattening pigs, but these changes did not reach statistical significance (*p* > 0.05, Table 5).

## 4. Discussion

This study provides integrated microbiota–metabolite evidence that fattening pigs supplemented with WE harbor a gut ecosystem functionally optimized for microbial community structure regulation, metabolic profile modulation, and immune-antioxidant enhancement, distinguishing them from the NC group. By jointly analyzing 16S rDNA sequencing and untargeted metabolomics data, we revealed how differences in microbial composition, differential taxa, metabolic output, and physiological indices collectively shape WE-induced intestinal strategies for health improvement.

### 4.1. Microbial Functional Optimization Without Diversity Changes

The optimization of microbial community function does not necessarily depend on diversity changes, a phenomenon confirmed here. Cui et al. [2] found tartary buckwheat flavonoids increased Bacteroidetes and Mitsuokella abundances without affecting α-diversity in piglet fecal microbiota, suggesting functional optimization may occur without diversity shifts. Although WE did not significantly alter microbiota α-diversity, its directional regulation of key functional bacteria (e.g., increased Firmicutes, decreased Proteobacteria) and coordinated improvements in metabolites and immune indices indicate microbiota restructuring toward “low inflammation and high metabolic efficiency.” This “quality optimization” may relate to polysaccharides in WE selectively promoting beneficial bacterial colonization [7]. Based on trends, WE increases beneficial bacteria and inhibits pathogens.

### 4.2. Gut Microbiota Remodeling: Shifts in Taxa and Functional Implications

It was previously shown that the predominant phyla in the fecal microbiota in fattening pigs are *Firmicutes*, *Proteobacteria*, and *Actinobacteriota* [15], which were also the most abundant microbial communities identified in this study. *Firmicutes* include a large number of fiber-degrading bacteria, which are the predominant microbiota in the gastrointestinal tract of animals and play a key role in the decomposition of fibrous feed [16]. In contrast, *Proteobacteria* is a phylum containing mostly harmful bacteria, including *Escherichia coli*, *Salmonella*, and *Vibrio cholerae*. A positive correlation between the abundance of *Firmicutes* and weight gain was reported [17], which aligns with our findings: compared to the NC group, the abundance of *Firmicutes* was relatively increased by 5.79%, and combined with our previous study [12], the average daily weight gain, carcass weight, and dressing percentage of fattening pigs in the WE group showed upward trends. The observed increase in *Firmicutes* abundance following WE supplementation may be attributed to the high polysaccharide content in WE (72.4%). As complex carbohydrates, polysaccharides are preferentially utilized by many *Firmicutes* species (e.g., *Lactobacillus*, *Clostridium*) as energy sources, promoting their proliferation [7]. These bacteria possess specific glycoside hydrolases and carbohydrate-active enzymes that enable efficient degradation of polysaccharides into fermentable monosaccharides and SCFAs, creating a nutrient-rich microenvironment that favors their growth over other phyla. Additionally, bioactive components in WE, such as polyphenols and flavonoids, may exert selective antimicrobial effects against competing phyla (e.g., *Proteobacteria*), indirectly enhancing the relative abundance of *Firmicutes* [10]. This synergistic effect of nutrient supply and selective inhibition likely contributes to the enrichment of *Firmicutes* in the WE group. Huang et al. [18] reported that increased abundance of *Proteobacteria* is likely a potential microbial marker of mammalian gut dysbiosis, which can trigger inflammatory responses and cause lesions [19]. In line with this, our data showed that *Proteobacteria* abundance was slightly decreased by 28.45% in the WE group compared to the NC group, accompanied by trends toward improved growth performance (average daily gain: +4.88%; carcass weight: +4.66%; dressing percentage: +4.82%) [12]. These results support the positive correlation between *Firmicutes* abundance and weight gain, but the effects of higher WE dosages or longer feeding cycles warrant further investigation.

At the genus level, *Lactobacillus* was the dominant microbial community in both groups, with the relative abundance of *Lactobacillus* in the WE group being 2.11% higher than that in the NC group. This change holds significant physiological importance: as a core beneficial bacterium in the intestine, *Lactobacillus* not only promotes intestinal digestion and absorption by decomposing nutrients such as carbohydrates, but also produces SCFAs (e.g., propionic acid) that serve as an energy source for intestinal epithelial cells. Moreover, it can induce intestinal epithelial cells to secrete antimicrobial peptides (e.g., defensins), enhancing the integrity of the intestinal mucosal barrier. The 1.09-fold upregulation of propionic acid in the WE group—consistent with the 2.11% higher *Lactobacillus* abundance (Supplementary Table S2)—contributes to improved intestinal barrier function by fueling epithelial cells, which aligns with the significant reduction in the intestinal inflammation-related metabolite MDA in the WE group. This is also consistent with previous studies showing that *Lactobacillus* enhances intestinal barrier function and reduces pathogenic invasion [20]. Meanwhile, the increased abundance of *Clostridium* in the WE group is noteworthy. As a key amino acid-metabolizing genus, *Clostridium* secretes proteases and peptidases to hydrolyze undigested proteins into absorbable small peptides and free amino acids, thereby enhancing nutrient utilization efficiency—a potential indirect mechanism linking *Clostridium* to growth performance [21]. In contrast, *Lactobacillus* ferments dietary carbohydrates to produce SCFAs (e.g., propionic acid), which directly improve nutrient absorption by serving as energy sources for intestinal epithelial cells [20]. Thus, the 2.11% increase in *Lactobacillus* abundance, though numerically small, may have functional significance: *Lactobacillus* acts synergistically with *Clostridium* and other beneficial bacteria to enhance nutrient utilization and reduce intestinal inflammation, as reflected by the 82.8% reduction in MDA (a marker of oxidative damage) and upward trends in average daily gain reported in our previous study [12]. Similar studies have shown that even moderate increases in *Lactobacillus* abundance can improve growth performance by modulating gut homeostasis and nutrient absorption efficiency [1,2].

In terms of pathogenic bacteria regulation, the abundances of *Shigella* and *Streptococcus* in the WE group decreased by 90.74% and 34.43%, respectively—changes crucial for intestinal health. *Shigella* is a major pathogenic bacterium causing intestinal infections and diarrhea, disrupting intestinal homeostasis by invading mucosal cells [22]; certain pathogenic *Streptococcus* strains induce intestinal inflammation by producing toxins such as hemolysin [23]. Their reduced abundance directly lowers the risk of intestinal infections and synergizes with upregulated anti-inflammatory metabolites (e.g., eicosadienoic acid) in the WE group to maintain intestinal microecological balance. Additionally, the abundance of *Turicibacter* (family *Erysipelotrichaceae*), a potential pathogenic bacterium associated with low-grade intestinal inflammation [24], showed a decreasing trend in the WE group, further confirming that WE improves intestinal health by inhibiting harmful bacterial colonization. In summary, WE constructs a benign cycle of “beneficial bacteria proliferation–optimized nutrient utilization–reduced inflammation risk” by directionally regulating intestinal flora: increasing functional bacteria (e.g., *Lactobacillus*, *Clostridium*) while inhibiting pathogens (e.g., *Shigella*, *Streptococcus*).

### 4.3. Mechanisms Underlying Flora Regulation: Roles of WE Bioactive Components

The regulatory effects of WE on intestinal flora and serum indices are likely mediated by its major bioactive components: polysaccharides (72.4%), total phenols (4.79 mg/g), and total flavonoids (0.07%). As the most abundant component, polysaccharides likely act as prebiotics—their complex structure is selectively fermented by beneficial bacteria such as *Lactobacillus* and *Clostridium* [7]. These bacteria use carbohydrate-active enzymes (e.g., glycoside hydrolases) to degrade polysaccharides into monosaccharides and SCFAs, providing energy for their own proliferation [20,21], explaining the increased abundance of *Lactobacillus* and *Clostridium* in the WE group. In contrast, phenolic compounds and flavonoids in WE may directly inhibit pathogens (e.g., *Shigella*, *Streptococcus*). Previous studies have shown that plant phenolics and flavonoids disrupt bacterial cell membranes, inhibit nucleic acid synthesis, and interfere with enzyme activity in Gram-negative pathogens like *Shigella* [10], aligning with the 90.74% reduction in *Shigella* abundance in the WE group.

These flora changes further impact serum indices through a “microbiota–metabolite–immunity” cascade. For instance, increased *Lactobacillus* and *Clostridium* promote SCFA production (e.g., propionic acid, upregulated by 1.09-fold), enhancing intestinal barrier function, and stimulating immunoglobulin synthesis (IgA, IgM, IgG; 3.97–4.67-fold increases) [20]. Meanwhile, reduced pathogenic bacteria and phenolic-mediated antioxidant effects contribute to the 82.8% decrease in serum MDA, a marker of oxidative damage. Collectively, WE components synergistically regulate flora structure, which in turn modulates serum immune and antioxidant indices—likely the core microbiological mechanism by which WE improves growth performance and intestinal health in fattening pigs. The trend-like improvement in growth performance (e.g., average daily gain) was inferred from associations with gut microbiota changes observed here, combined with our previous findings [12] where 0.1% WE supplementation increased average daily gain by 4.88%. However, growth performance was not directly measured in the current study, so this conclusion remains indirect and requires further validation.

### 4.4. Metabolic Reprogramming: Key Metabolites and Pathways

In this study, untargeted metabolomics identified fecal metabolite differences between the NC and WE groups. To ensure accuracy, we integrated VIP values from the OPLS-DA model with FDR correction—an approach that minimizes false positives in multivariate analysis and provides biologically meaningful interpretations compared to single *p*-value screening [1]. The stringent criterion of FDR < 0.05 further validated metabolite differences, aligning with methodological standards of similar research [2].

Dietary WE significantly altered the fecal metabolite profile of fattening pigs. Notably, phosphatidylcholine PC (18:3/18:3) and propionic acid were upregulated by 3.08-fold and 1.09-fold, respectively (Table 3), with critical roles in lipid metabolism regulation. PC (18:3/18:3), a major component of lipid droplet membranes, is closely linked to fat deposition [25]; its increased content may directly participate in adipocyte differentiation and triglyceride synthesis, providing a structural basis for intracellular lipid storage. As a key product of intestinal flora metabolism, propionic acid not only promotes absorption of fats and proteins in the small intestine but also serves as a precursor for gluconeogenesis and lipogenesis [26]. Additionally, it improves the intestinal environment through feed acidification, indirectly enhancing growth performance [27,28,29]. The upregulation of these metabolites aligns with our current and previous results: although serum TG, TC, and LDL-C showed no significant changes in the WE group, trends toward increased TG (+36.0%) and TC (+11.6%), combined with significant increases in backfat thickness (+33.12%) and intramuscular fat content (+98.59%) in previous work [12], suggest that WE enhances directional lipid deposition (rather than systemic accumulation) via upregulation of PC (18:3/18:3) and propionic acid. Furthermore, KEGG pathway analysis showed significant enrichment of glycerophospholipid metabolism and fat digestion/absorption pathways (Figure 5C), confirming that WE promotes efficient lipid utilization through these metabolites—improving meat quality-related traits like intramuscular fat without causing abnormal serum lipid elevation, a feature valuable for meat quality improvement and metabolic disorder prevention.

### 4.5. Anti-Inflammatory and Antioxidant Effects: Metabolite–Microbiota Synergy

WE significantly improved intestinal health in fattening pigs by regulating synergistic interactions between specific metabolites and gut microbiota, via three core mechanisms:

Targeted inflammatory pathway regulation: WE exerts anti-inflammatory effects by upregulating metabolites that target key pathways. As an ω-6 polyunsaturated fatty acid, eicosadienoic acid (2.05-fold upregulated in the WE group) may reduce pro-inflammatory factor release by inhibiting the NF-κB pathway [30,31], suggesting a role for ω-6 PUFAs in modulating intestinal inflammation (though higher dosages or longer interventions may be needed to confirm effect magnitude). Additionally, methyl cinnamate (0.79-fold upregulated) alleviates intestinal inflammation by inhibiting the MAPK pathway [32,33]. Combined with increased *Firmicutes* (5.79%) and *Lactobacillus* (2.11%) abundances—both positively correlated with methyl cinnamate levels—this suggests synergism between methyl cinnamate and Lactobacillus, consistent with previous studies showing that methyl cinnamate improves gut dysbiosis in DSS-induced colitis mice by inhibiting the MAPK pathway and increasing *Firmicutes*/*Lactobacillus* abundance [34,35]. Supporting evidence includes the following: *Lactobacillus* metabolites inhibit MAPK activation [1,2], synergizing with methyl cinnamate; methyl cinnamate reduces *p*-ERK and *p*-p38 phosphorylation in colitis models [3]; and KEGG analysis of WE-treated pigs shows significant MAPK pathway enrichment, linking metabolic changes to inflammatory signaling [4]. We speculate they synergistically regulate MAPK signaling, but direct evidence from MAPK component protein/gene expression is needed. Microbiota-mediated inflammation regulation: The synergistic effect of methyl cinnamate and *Lactobacillus* strengthens microbial regulation of inflammation, as discussed above.

Antioxidant capacity enhancement: WE improves intestinal health by upregulating antioxidant metabolites. SOD activity increased by 4.58% and MDA content decreased by 82.8% in the WE group, indicating enhanced antioxidant capacity. Upregulated glutamine—a key precursor for glutathione synthesis—likely contributes to these effects, particularly the 82.8% reduction in serum MDA (a marker of oxidative damage). Glutathione, synthesized from glutamine, scavenges free radicals, aligning with improved redox balance in the WE group. While GSH-Px activity showed no significant difference, this may reflect the short experimental period or glutamine upregulation magnitude (sufficient to reduce MDA but not yet affect GSH-Px detectably). In summary, WE synergistically improves intestinal health through these mechanisms, while upregulated PC (18:3/18:3) and propionic acid promote directional lipid deposition—providing a clear theoretical basis for WE as a functional feed additive.

### 4.6. KEGG Pathway Enrichment: Linking Metabolism to Immunity and Lipid Regulation

KEGG pathway enrichment analysis of differentially expressed metabolites revealed significant upregulation of several inflammation-related pathways in the WE group, including glycerophospholipid metabolism, choline metabolism, linoleic acid metabolism, and α-linolenic acid metabolism. Glycerophospholipid metabolism is key to systemic immune regulation and low-grade inflammation [36], while choline supplementation alleviates intestinal inflammation in weaned piglets by modulating gut microbiota [37], suggesting WE may synergistically enhance intestinal immune barrier function via these pathways. Additionally, the linoleic acid derivative 9,12,13-TriHOME has confirmed anti-inflammatory effects [38], and α-linolenic acid (an essential fatty acid) inhibits inflammatory signaling [39]; activation of these pathways further strengthens WE’s anti-inflammatory potential. This aligns with significantly increased IgA, IgM, and IgG levels in the WE group, indicating that WE may promote immunoglobulin synthesis via these pathways to enhance immune responses and intestinal health.

Differentially accumulated metabolites were also enriched in lipid metabolism-related pathways, including arachidonic acid metabolism [40], glycerophospholipid metabolism, linoleic acid metabolism [41], glycerolipid metabolism, lipolysis regulation [42], and digestion/absorption pathways. The a ctivation of these pathways indicates that WE enhances lipid metabolism capacity in fattening pigs—providing energy and participating in immune regulation via lipid mediators (e.g., arachidonic acid derivatives, unsaturated fatty acids)—forming a “lipid metabolism–immune function” synergistic network. This explains how WE improves growth performance and intestinal health.

### 4.7. Oxidative Stress and Amino Acid Metabolism

Oxidative stress is a pathogenic factor in intestinal inflammation [43], with decreased SOD, GSH-Px, CAT, and T-AOC levels, and increased serum MDA, associated with inflammatory onset and progression. Notably, WE supplementation significantly reduced serum MDA (−82.80%), indicating improved antioxidant capacity. Although SOD, GSH-Px, CAT, and T-AOC activities trended upward, these changes were not statistically significant—potentially due to the short experimental period (40 days) or need for higher WE dosages.

Metabolomics sequencing revealed most differentially accumulated metabolites were amino acids and their derivatives. These exhibit antioxidant properties via sulfur or amino groups, participating directly/indirectly in antioxidant processes. For example, glycine and glutamate are components of glutathione (a major hepatic antioxidant) [44]; glutamine (a glutathione precursor) helps cells resist oxidative stress, scavenge free radicals, enhance immune responses, reduce inflammation, and modulate antioxidant/anti-inflammatory pathways [45]. In the WE group, most amino acids and small peptides were more abundant than in the NC group, consistent with antioxidant indices—indicating amino acid metabolism partially contributes to enhanced antioxidant capacity in WE-supplemented pigs.

Polysaccharides—natural nutrients with immune-enhancing, anti-inflammatory, antioxidant, and gut health-promoting properties [46,47,48]—constitute 72.4% of WE. In this study, WE improved immune and antioxidant indices, with multiple metabolites and pathways linked to anti-inflammation—effects closely associated with its polysaccharide content. Overall, WE enhances antioxidant and immune functions in fattening pigs, likely via combined actions of polysaccharides and amino acid metabolism regulation, though detailed mechanisms require further investigation.

### 4.8. “Microbiota–Metabolite–Immunity” Cascade Regulation

From the perspective of “microbiota–metabolite–immunity” cascade regulation, WE drives this functional axis through stepwise, coordinated microbial shifts, metabolic reprogramming, and immune modulation. Microbial–metabolite signaling: WE first enriches beneficial bacteria such as *Clostridium*, whose increased abundance coincides with upregulated fecal propionic acid (a pivotal SCFA with well-characterized immunomodulatory roles). *Lactobacillus* (2.11% more abundant) reinforces this cascade by fermenting dietary fiber to produce SCFAs (including propionic acid), which support intestinal barrier integrity as epithelial energy substrates and inhibit pro-inflammatory cytokine release via GPR43 activation [20,27], bridging microbial activity to immune homeostasis. Lipid metabolism branch: The 5.79% increase in *Firmicutes* abundance is tightly coupled with elevated PC (18:3/18:3)—a key phosphatidylcholine—reflecting microbial control over lipid pathways to regulate energy homeostasis [25,26]. Functionally, PC (18:3/18:3) is integral to immune cell membrane synthesis, supported by the 4.43-fold increase in serum IgG, highlighting how microbial-driven lipid metabolites fuel antibody-producing cells. Immune and antioxidant outcomes: These changes collectively enhance immune and antioxidant responses: 3.97-fold higher IgA, 4.43-fold higher IgG, and 82.84% lower MDA align with upregulated propionic acid and methyl cinnamate. Propionic acid reduces inflammatory mediators via NF-κB inhibition and promotes antioxidant enzyme expression [28,45], linking microbial metabolites to improved redox balance. Methyl cinnamate (1.73-fold upregulated) synergizes with increased *Lactobacillus* to strengthen intestinal barrier function via MAPK inhibition [34,35], further dampening inflammation and boosting immunity. KEGG pathway analysis validates these interactions: WE intervention enriches glycerophospholipid and α-linolenic acid metabolism, where key metabolites (e.g., PC, eicosadienoic acid) are microbiota-regulated and exert immune effects via lipid signaling. For example, α-linolenic acid metabolites—with metabolic flux correlating to *Firmicutes* abundance [39,41]—inhibit inflammatory signaling, reinforcing links between microbial activity and immune regulation.

## 5. Conclusions

Walnut green husk extract (WE) is rich in polysaccharides. This study found that the addition of 0.1% WE to the diet optimized the gut microbiota structure without significantly altering microbial diversity. Specifically, it increased the abundance of beneficial bacteria (e.g., *Firmicutes*, *Lactobacillus*) while reducing the abundance of pathogenic bacteria (e.g., *Proteobacteria*, *Shigella*). Combined with metabolomics sequencing results, the upregulation of organic acids and their derivatives, as well as amino acids and their metabolites, significantly enhanced immune and antioxidant levels in fattening pigs. Additionally, WE modulated lipid metabolism and inflammation-related pathways, contributing to improved gut health. These findings suggest that WE has the potential to replace antibiotic growth promoters, effectively enhancing the production performance and meat quality of fattening pigs. This study not only provides a scientific basis for the resource utilization of walnut processing by-products but also offers new insights for developing eco-friendly functional feeds and promoting sustainable livestock production. Future research could further explore the effects of WE at different doses and under long-term use to fully unlock its potential applications.

## Figures and Tables

**Table 1:** Composition and nutrient levels of the basal diet ^1^ (air-dried; %).

Ingredients	Content
Corn	58.00
Soybean meal	14.00
Wheat bran	9.00
Grass meal	15.00
Premix ^2^	4.00
Total	100.00
Nutrient levels ^3^	
Digestive energy/(MJ/kg)	11.33
Crude protein	13.13
Crude fiber	9.10
Crude ash	8.30
Calcium	0.60
Phosphorus	0.50
Lysine	0.90

^1^ The basal diets were formulated according to the *Nutrient Requirements of Swine* (NRC, 2012) and the Chinese Feeding Standard for Swine (NY/T 65-2004) [14]. ^2^ The premix provided the following per kg of feed: 10,800 IU of VA, 10 mg of VB_1_, 3000 IU of VD_3_, 80 mg of VE, 3000 IU of VK_3_, 20 mg of VB_12_, 200 mg of biotin, 15 mg of D-pantothenic acid, 10 mg of nicotinic acid, 90 mg of Fe (as ferrous sulfate), 25 mg of Cu (as copper sulfate), 100 mg of Zn (as oxide zinc), and 15 mg of Mn (as manganese sulfate). ^3^ The digestive energy was determined as a calculated value, while the others were measured values.

**Table 2:** Adonis test analysis of the effects of dietary walnut green husk extract on the fecal microbial community structure in fattening pigs.

Type	Df	Sum of Squares	*R^2^*	*F*	*p*-Value
Group	1	0.005001	0.046042	0.193057	1.000000
Residual	4	0.103613	0.953958	—	—
Total	5	0.108614	1.000000	—	—

**Table 3:** Changes in key differential metabolites in feces of fattening pigs.

Compounds	VIP	*p*-Value	FDR	Log_2_FC	Type
PC(18:3/18:3)	1.90	0.05	0.56	3.08	up
[(2S)-1-decanoyloxy-3-hydroxypropan-2-yl] icosanoate	1.98	0.00	0.07	2.70	up
Gln-Leu-Leu	1.88	0.05	0.56	2.50	up
N-(dodecanoyl)-sphing-4-enine-1-phosphocholine	1.70	0.01	0.28	2.28	up
9,11-methane-epoxy Prostaglandin F1alpha	1.72	0.00	0.25	2.25	up
(2S,4S)-1-Acetoxy-16-heptadecene-2,4-diol	1.95	0.00	0.09	2.17	up
Palmitic amide	1.85	0.00	0.19	1.96	up
Gentian violet	1.80	0.03	0.50	1.88	up
Propionic acid	1.68	0.03	0.52	1.09	up
Eicosadienoic acid	1.63	0.04	0.53	1.04	up
Methyl cinnamate	1.96	0.01	0.34	0.79	up
cis-Annonacin-10-one	2.00	0.00	0.16	−9.91	down
PC(14:0/18:4(6Z,9Z,12Z,15Z))	1.97	0.00	0.23	−7.47	down
1-O-Hexadecyl-2-arachidonoyl-sn-glycero-3-phosphocholine	1.81	0.00	0.07	−4.28	down
PC(18:3(9Z,12Z,15Z)/18:3(9Z,12Z,15Z))	1.75	0.00	0.19	−4.23	down
2′-Deoxyuridine 5′-monophosphate	1.99	0.00	0.14	−4.14	down
Panaxatriol	1.98	0.02	0.43	−2.49	down
PC(15:0/18:3(6Z,9Z,12Z))	1.67	0.00	0.19	−2.22	down
Ditrans, polycis-undecaprenyl phosphate	1.63	0.02	0.43	−2.04	down
Canthaxanthin	1.54	0.01	0.31	−1.94	down
PE-NMe(15:0/22:2(13Z,16Z))	1.64	0.01	0.25	−1.92	down
Arachidonic Acid Leelamide	1.10	0.03	0.52	−1.64	down

**Table 4:** Effect of dietary supplementation with walnut green husk extract on serum antioxidant activities in fattening pigs.

Items	NC	WE	*p*-Value
SOD, U/mL	66.44 ± 2.83	69.48 ± 3.30	0.50
GSH-Px, U/mL	671.16 ± 17.69	676.50 ± 37.78	0.91
CAT, U/mL	102.45 ± 12.17	124.50 ± 8.08	0.20
MDA, nmol/mL	4.72 ± 0.43	0.81 ± 0.15	<0.01
T-AOC, U/mL	10.29 ± 3.09	18.42 ± 2.91	0.09

Note: SODS, superoxide dismutase; GSH-Px, glutathione peroxidase; CAT, catalase.

**Table 5:** Effect of dietary supplementation with walnut green husk extract on serum lipid indices in fattening pigs.

Items	NC	WE	*p*-Value
TG, mmol/L	0.50 ± 0.01	0.68 ± 0.10	0.18
TC, mmol/L	2.32 ± 0.20	2.59 ± 0.07	0.19
LDL-C, mmol/L	1.42 ± 0.18	1.22 ± 0.18	0.47
HDL-C, mmol/L	1.01 ± 0.04	1.01 ± 0.08	0.99

Note: TC, total cholesterol; TG, triglycerides; HDL-C, high-density lipoprotein cholesterol; LDL-C, low-density lipoprotein cholesterol.

## Data Availability

The data presented in the study are deposited in the figshare database (https://doi.org/10.6084/m9.figshare.27643830, https://doi.org/10.6084/m9.figshare.27916782).

## Supplementary Materials

The following supporting information can be downloaded at https://www.mdpi.com/article/10.3390/ani15162395/s1, Table S1: The abundance of fecal microbiota in fattening pigs at phylum level; Table S2: The abundance of fecal microbiota in fattening pigs at genus level; Table S3: Information on metabolites detected in different ion modes; Table S4: Types of differentially accumulated metabolites; Table S5: Number of differentially accumulated metabolites class.
